# Toward a dynamical understanding of microbial communities

**DOI:** 10.1098/rstb.2019.0248

**Published:** 2020-03-23

**Authors:** Paul B. Rainey, Steven D. Quistad

**Affiliations:** 1 Department of Microbial Population Biology, Max Planck Institute for Evolutionary Biology, 24306 Plön, Germany; 2 Laboratoire de Génétique de l'Evolution, Chemistry, Biology and Innovation (CBI) UMR8231, ESPCI Paris, CNRS, PSL Research University, 75231 Paris, France

**Keywords:** eco-evolutionary dynamics, lateral gene transfer, experimental community evolution, metagenomics

## Abstract

The challenge of moving beyond descriptions of microbial community composition to the point where understanding underlying eco-evolutionary dynamics emerges is daunting. While it is tempting to simplify through use of model communities composed of a small number of types, there is a risk that such strategies fail to capture processes that might be specific and intrinsic to complexity of the community itself. Here, we describe approaches that embrace this complexity and show that, in combination with metagenomic strategies, dynamical insight is increasingly possible. Arising from these studies is mounting evidence of rapid eco-evolutionary change among lineages and a sense that processes, particularly those mediated by horizontal gene transfer, not only are integral to system function, but are central to long-term persistence. That such dynamic, systems-level insight is now possible, means that the study and manipulation of microbial communities can move to new levels of inquiry.

This article is part of the theme issue ‘Conceptual challenges in microbial community ecology’.

## 1. Introduction

No matter one's perspective, microbial communities are mind-bogglingly complex [1–3]. The sense of complexity comes not just from the vast number of often densely packed cells [4,5], or the genetic, physiological and functional diversity of types [6–8], but particularly from the recognition that cells in close proximity compete and interact [9,10]. The range, scale and dynamic of these interactions are largely unknown. Attempts to comprehend the multiplicity of effects, including those on the patterning of diversity, addle the mind.

But the challenge is even more acute: ecology is only part of the picture. Ecological processes determine the selective conditions that drive evolutionary change, which in turn feed back to affect patterns of diversity, which prompt further evolutionary change in iterative and continual cycles [11,12]. But what is the dynamic of this process? Over what spatial and temporal scales should the effects be measured? How can the effects even be measured? Does evolution matter, or is it all just ecology? How does the field move beyond description of types to the point where insight emerges into the feedback between ecological and evolutionary process—ideally with links to function? How do we even decide what matters? Is it conceivable that community function is shaped by processes that are intrinsic to community complexity?

At one level, answers to most of these questions are apparent and we do not wish to downplay the enormous advances that have come from the ability to describe community composition [13,14], or from the use of simplified model communities composed of representative types [12,15,16], including interactions [17,18], but here we aim to advocate thinking about communities as dynamic systems. Such a view places emphasis on process, on the connection between process and function, and on the connection between process and ecological opportunity. It brings to the fore eco-evolutionary feedbacks between genes, the phenotypes they determine, and the effects wrought by selection acting on phenotypic differences among the populations that compose communities and even ecosystems [11]. Ultimately, it raises the possibility that microbial communities might be shaped by processes, such as that mediated by horizontal gene transfer (HGT), that while recognized at the population level, assume, in the context of communities, a dynamic and impact that remain under-appreciated. If true, then simplification, for example through use of synthetic or model communities—typically such a powerful way of doing science—may fail to capture community-level processes.

## 2. Dynamics from genomics and metagenomics

One strategy is to adopt standard population genetic approaches and apply these to the analysis of communities. Such approaches allow the drivers of evolutionary change to be deduced from patterns of diversity. For example, the relative contributions of mutation, recombination, migration, selection and genetic drift can be inferred from patterns of allelic variation provided by DNA sequence data [19]. From such data, and via the tools of population genetics, it is even possible to predict future changes in patterns of diversity [20]. The kind of data and routes to analysis are well established for individual populations and species (see for example [21]), but an extension to complex microbial communities is in its infancy.

Two kinds of information are important. The first concerns the traits under selection, and the second concerns evolutionary processes affecting the spread of mutants, both within and between communities. Work using comparative genomics and model organisms has shed light on the traits under selection in communities ranging from the gut microbiome [22] to the CF lung [23], to endosymbiotic communities of sponges [24] and communities of microbial mats [25].

Data providing insight into processes affecting the spread of mutants within the context of communities are not so easily acquired. Much is in principle possible via whole-genome sequence analysis of communities (metagenomics); however, at the present time most metagenomic data come from high-throughput sequencing platforms such as Illumina that deliver an abundance of sequence reads, but of typically short length (in the range of 150–300 bp). Such data are difficult to deal with from a population genetic perspective, because of uncertainty over the linkage between independent reads. For example, a metagenomic dataset that shows a high level of mismatch when reads are aligned to a given reference genome may derive from a community that contains a single strain, or multiple divergent strains [26].

Advances in computational approaches have led to improved abilities to detect variants within individual species [27–29] and even to identify individual strains [28,30] within complex datasets, but only very recently have analyses emerged that provide insight into the dynamic of the evolutionary process.

A recent study [31] focused on the most abundant strains from gut microbiomes and limited inquiries to hosts with lineages of bacteria whose genetic structure was simple so that it was possible to assign alleles to a dominant lineage with high confidence. With focus on 40 prevalent species from hundreds of previously published human stool metagenomes—with some samples taken at multiple time points—Garud *et al.* [31] drew numerous inferences concerning evolutionary and ecological dynamics. Many findings are worth noting. Firstly, evidence was provided of both short- and long-term evolutionary change. This is important because it says that evolution in microbial communities really does happen: evolutionary and ecological processes shape patterns of diversity in the microbiome—and by extension in microbial communities more generally. This finding fits with studies using culturable focal organisms that we mention briefly below [32,33]. Secondly, they showed changes in patterns of sequence diversity indicative of local adaptation on short timescales, but also extinction events over longer timescales, with the latter mediated by migration from different hosts.

Thirdly, and perhaps most intriguingly, the data of Garud *et al.* [31] did not sit in full accord with expectations from simple null models from population genetics. This supports the idea that eco-evolutionary dynamics in microbial communities may be influenced by community-level processes—particularly HGT—whose effects are not simply an extension of what we have come to understand from the study of populations and species [34,35].

Despite caveats and uncertainties, the work of Garud *et al.* [31] shows that dynamical processes within natural microbial communities do occur, that they can be quantified and that quantification can lead to reliable inferences. With advances in single-molecule sequencing technologies now yielding single reads in excess of 10 kilobase pairs (kbp) (and as high as 2 Mbp), combined with bioinformatic pipelines for analysis of metagenomic data [36], there is every reason to expect community-level genomics to lead to the kinds of insights that genomics has delivered for the analysis of populations and species.

Uncertainties that stem from sole reliance on metagenomic data can to some extent be overcome by combining genome sequence from individual (culturable) isolates with metagenomic data. Zhao *et al.* [33] explored within-host evolution of *Bacteroides fragilis*, a commensal from the large intestine of humans. They began with isolation and sequencing of more than 600 individual *B. fragilis* isolates from stool samples from 12 individual humans. While much was learned from the analysis of these genomes, including the fine-scale dynamics of within-host diversification, insight into broader community-level dynamics, and particularly impact of the community of gut microbes on evolution of the focal isolates, came from mapping metagenomic data—acquired from the same human subjects—onto the 600 *B. fragilis* genomes.

From such mapping, Zhao *et al.* were able to demonstrate the transfer of mobile genetic elements from the broader community, into specific focal strains. Additionally, using the same approach, but combined with densely sampled metagenomic time series from a subset of subjects, the authors were able to investigate evolutionary dynamics at a high level of temporal resolution. This provided evidence of the coexistence of multiple *B. fragilis* sub-lineages, which prompted laboratory-based experiments to explore the stability of the observed polymorphisms.

Interestingly, findings from *in vitro* work did not recapitulate observations derived from the metagenomic data. As the authors point out, such findings draw attention to the difficulties of reconstructing simplified microbial communities *in vitro*. To us, there seems little *a priori* reason to suspect that what happens in artificial medium devoid of community context will recapitulate multipartite interactions that underpin eco-evolutionary dynamics in natural communities [37]. This forcefully emphasizes the power of metagenomics, especially when combined with the reference genomes, to provide insight into dynamical processes affecting the fate of microbes in complex and diverse communities.

## 3. Dynamics from meta3C

A typical metagenomic study delivers millions of short DNA sequence reads that, depending on the depth of coverage, might be assembled into hundreds of thousands of contiguous sequences (contigs), with an average contig size in the kilobase pair range. Knowledge of contig proximity is largely absent and thus ability to assemble them into genomes is limited. When the community comprises a diverse range of species (each composed of numerous lineages), then even mapping to reference genomes (where they exist) may fail to improve matters. Many of the difficulties would be solved if it were possible to assemble contigs into complete genomes. A technique known as 3C (chromosome conformation capture), or Hi-C, does precisely this, but additionally provides quantitative data on interactions among DNA molecules. The latter in particular stands to revolutionize understanding of dynamical processes within communities.

The technique (3C) measures physical contacts by DNA segments within and between DNA molecules and has been used extensively to analyse the three-dimensional structure of genomes [38]. We refer the reader to specialized literature for detailed protocols (or for a review, see [39]), but in brief, contacts are frozen in time by the addition of a cross-linking agent. The trapped DNA is then digested with a restriction endonuclease and the cut fragments then re-ligated. This results in a strong bias toward fragments that, prior to digestion, were physically close and thus likely to share the same cellular compartment. The frequency distribution of events is determined by paired-end sequencing. Knowledge of the distribution, combined with the fact that the distribution follows the laws of polymer physics, is then used to identify synteny between DNA fragments up to several hundred kilobase pairs apart.

Koszul and colleagues [40] recognized that 3C had utility beyond studying the structure of individual chromosomes and could be applied to metagenomic data (they coined the term meta3C) to not only assemble contigs into chromosomes, but also capture interactions between chromosomes and accessory elements such as bacteriophages and plasmids. The latter can be further used to identify the range of chromosomes (and thus cells) that such elements co-associate with and even the temporal dynamic of associations. For example, a plasmid that exists in cell A and also cell B will show evidence of interaction (DNA–DNA contact) with the chromosomes of both A and B. With temporally- and time-resolved data the dynamic of the association can be measured.

That it is possible to track mobile genetic elements within complex communities by capturing DNA contacts within and between DNA molecules was powerfully demonstrated by applying meta3C to the mouse gut microbiome [41]. From a single meta3C library Marbouty *et al.* [41] were able to assemble numerous bacterial chromsomes and bacterio phages and to identify the association between bacterio phages and their respective bacterial hosts.

In a recent study, Yaffe & Relman [42] took the same meta3C approach coupled with a probabilistic modelling framework to resolve 88 genomes from human gut microbiomes and thousands of accessory elements. Integral to the study were samples from two individuals spaced by an interval of 10 years. Armed with this temporal dimension, the authors quantified the evolutionary fate of elements over the 10-year period, the extent of selection on the core genomes, and eco-evolutionary dynamics of the accessory elements, and even measured rates of HGT. The latter, 4–19 genes per year, exceeded core site substitution rates for the majority of genomes.

This level of insight from culture-independent and reference genome-free strategies is impressive. The door to further discovery is now wide open, with these studies already providing a glimpse of dynamical processes within complex communities—especially those associated with mobile genetic elements and HGT—that are likely fundamental to how microbial communities function and evolve. In fact, evidence of substantive movement of DNA between community members is the essence of the black queen hypothesis [43,44] and likely plays a significant role in distributing genes necessary for community function among cellular lineages [45].

## 4. Experimental manipulation of dynamics

A complementary approach to those involving direct observation is to impose some kind of experimental manipulation on a given community, ideally affecting a single evolutionary (or ecological) process, and to then determine the effect of the manipulation both on the underlying eco-evolutionary dynamics of community members and on community function. We mention one such manipulation that derives from our own recent work. The research article appears elsewhere in this special issue [46]; thus we provide only brief mention here.

Inspiration for the experiment came in part from the power and elegance of selection experiments with single genotype microbes, where evolutionary change arising after a period of serial propagation can be attributed primarily to mutation and selection [47]. As discussed above, a process of significance for microbial communities—and which may have community-level properties, dynamics and effects—is the transfer of genetic material between often distantly related bacteria by bacteriophages and other selfish genetic elements (SGEs) [48,49]. A rough analogy might equate such a process with sexual reproduction within species.

Studies of the evolutionary consequences of sex have been most powerfully analysed via experiments in which the evolutionary performances of isogenic sexual and asexual types have been compared. While not possible in most species, the genetic tractability of yeast delivered the necessary strains, and analysis of the rate at which each type adapted to new environments showed that sex enhances the pace of adaptive evolution [50,51].

Given that sex has this impact on the evolutionary dynamics of species—along with numerous other consequences—what impact might lateral gene transfer have on communities? Could the process be manipulated in a manner akin to the yeast experiment? Drawing upon theory surrounding SGEs [52–54], long-term persistence of such elements requires periodic exposure to new hosts [55]. In the absence of such exposure, and thus an opportunity for transfer, the selection is powerless to prevent loss of transfer ability. That loss occurs is evidenced by the remnants of prophages that litter bacterial genomes [56,57]. Conversely, frequent exposure to new hosts is expected to breathe evolutionary life into SGEs.

Accordingly, experimental communities comprising hundreds of microbial genera were established from independent samples of garden compost and maintained on paper (cellulose) as a sole carbon source with bi-weekly serial transfer. After establishment, each community was divided in two, with one subject to ‘vertical’ and the other to ‘horizontal’ transfer of SGEs. Vertical transmission was achieved simply by serial dilution. For the horizontal treatment, SGEs were collected from each of the horizontal communities, pooled and then redistributed among all horizontal communities. The experiment was maintained for 1 year (for details see [46]).

During the course of the experiment, DNA samples were collected at regular intervals from all mesocosms and subjected to metagenomic analyses. As evident in the accompanying paper [46], the horizontal treatment expected to fuel life of SGEs was highly effective at moving not just SGEs, but also genes of ecological significance. In fact, just two weeks following the division of communities into horizontal and vertical treatments, 17% of all sequence reads (from a total of 2.6 million reads per mesocosm) were derived from allopatric communities. On assembly, these allopatric sequence reads produced an average of approximately 3300 contigs greater than 1 kb per horizontal mesocosm. This extraordinary flux of DNA between communities is clear evidence of a highly dynamic process.

By read-mapping metagenomic data from time points taken throughout the experiment back on reference genomes from phages and other mobile DNA obtained at the start of the experiment, it was possible to capture the dynamics of amplification and dissemination. Moreover, analysis of horizontally transferred DNA revealed the movement of genes predicted to contribute to community function. These included genes implicated in the degradation of cellulose, iron scavenging and nitrogen metabolism. This prompted functional assays of ammonification, which showed significantly more ammonia in mesocosms in which SGEs transferred horizontally, compared with mesocosms in which SGEs were deprived of encountering new hosts, thus linking genes to community function.

Although scraping just the surface of community complexity, this experimental approach demonstrates that it is possible to study complex and hugely diverse communities in a dynamical way, akin to a researcher interested in the evolutionary and ecological implications of sex. Moreover, the experimental strategy can be readily applied elsewhere. For example, glass mesocosms containing paper could be substituted by mice. Further insight stands to come from the application of metagenomic strategies targeting mRNA [58]. Additional possibilities arise from spiking communities with specific genotypes whose dynamics are tracked at high resolution using bar-coding in conjunction with periodic genome re-sequencing [59]. The latter in particular allows the impact of community on the evolution of individual genotypes to be determined [32,33]. Further insight into dynamics at a very high level of resolution stands to come from the application of meta3C technologies as described in the section above (§3).

## 5. Dynamic interactions

In the spirit of embracing microbial community complexity, one additional and potentially fruitful route to progress involves top-down engineering of communities through manipulations that cause communities to become units of selection in their own right [60–67]. Such manipulations have immense practical applications in medicine, agriculture and biotechnology [61], but additionally provide a route to study the evolution of interactions that underpin community stability and function [67,68].

The idea that communities can be manipulated as units of selection was articulated 30 years ago [60] and realized in various experimental forms over ensuing years [64,65,69]. The idea is simple and draws upon the logic of Darwinism: entities, for example, communities of cells, will participate in the process of evolution by natural selection provided the communities vary one from another, communities replicate, and offspring communities resemble parental types. An additional requirement is that at least some of the variation between communities be linked to replicative success [70,71].

Researchers can readily confine communities to discrete patches, for example, by placing independent communities in separate test tubes, flasks, mice, insects, or similarly bounded structures. After a period of cell growth, community performance can be assessed using some assay of function [72]. This may even involve assay of the metaorganism, which combines microbial community and host organism. On the basis of the assay results, poor-performing communities are marked for extinction, whereas remaining communities are diluted and allowed to reproduce. Reproduction occurs by allowing successful communities to establish more than a single new community. This process, in which (artificial) selection acts on lineages of communities, can generate surprising effects. This comes from the fact that selection operates on at least two timescales: the doubling time of individual cells and the doubling time of communities [63,69,73]. Because success or failure of communities over the longer timescale depends on the function of community members, selection rewards those communities whose cells contribute toward community function: selection over the longer timescale stands to trump short-term within-community selection.

While both variation among communities and reproduction of communities are readily achievable, the extent to which offspring communities resemble parental communities is, at least initially, much less certain. Consider for a moment a hypothetical community composed of 10 different species that exceeds a measure of community-level performance provided each species is present at an approximately equivalent ratio. Such a community is thus selected and divided in two, giving rise to two offspring communities. Assuming no interactions among cells, stochastic effects of sampling arising during the dilution phase make it unlikely that the newly founded communities will recapitulate the parental community. Accordingly, the two offspring communities likely face extinction after the next assay. Consider then, the evolution of interactions among constituent species that improve the likelihood that the parental phenotype is recapitulated: a community with such capacity will, therefore, spread as a consequence of community-level selection. The process of community-level selection thus favours the evolution of interactions that increasingly align the reproductive fate of cells with that of the community [67]. Taken to the extreme, derived communities, after many generations of community-level selection, are likely to become organism-like, rather like insects and their symbionts, or the eukaryotic cell that evolved from a community of once independently replicating archaebacterial- and eubacterial-like cells [60].

Treatment of communities as units of selection thus offers the opportunity not only to engineer communities, but to observe the evolution of interactions, the dynamic of which can be followed using the tools and strategies of metagenomics and associated technologies discussed above. In addition to insight into dynamics, the tools of genetics, molecular biology and biochemistry can be applied to deconstruct—and in future, recapitulate—events leading to the emergence of communities capable of participating directly in the process of evolution by natural selection as units in their own right and with the necessary Darwinian properties being endogenously determined by the communities themselves.

## 6. Conclusion

Mounting evidence supports the view that microbial communities are complex dynamic systems. Interactions among an immense diversity of types shape the relationship between cells and their biotic and abiotic environment. These myriad of interactions—which are not hardwired—establish feedbacks between the environment each cell experiences and determines the future eco-evolutionary response of those cells and descendant lineages. This acts to further change the nature of interactions, provoking continual change—and change that maybe further influenced by the migration of communities between environments [34]. The challenge of studying such dynamic systems is considerable.

Here, we have advocated approaches that embrace this complexity. The reason for doing so stems from our sense that microbial community function depends on processes that are intrinsic to the system itself: simplify, via, for example, analysis of the interactions among a small number of focal genotypes maintained under laboratory conditions, and the opportunity to detect and quantify processes that define the system may well be lost. On one level this is trivial: if the nitrogen cycle requires 10 species to operate, then a two-fold reduction of diversity will clearly eliminate a given community's capacity to cycle nitrogen. But there is arguably a further and analogous layer of functional significance that embraces ecologically significant genes moved by HGT.

There are parallels between this view and that recently advocated by Doolittle and colleagues [35,74], the so named ‘it's the song not the singer’ (ITSNTS) theory. ITSNTS argues the importance of recognizing that selection can act on processes leading to persistence of, for example, microbial communities, even though such communities are for the most part not participating directly in the process of evolution by natural selection—at least not as units in the sense of Lewontin [70]. By encompassing a process-focused view, attention shifts to the nature of the process that sustains function. In the context of microbial communities, a process with the capacity to ensure persistence of function, and that is arguably shaped by selection, is HGT. The movement of genes between individuals in diverse communities serves to promote convergence upon shared function provided by diverse components [45]. Taking this a further step, there exists the intriguing possibility that the genetic information moved between entities defines a community-level interaction network that achieves a dynamic and functional effect that vastly exceeds the sum of the component HGT events.

There are important implications that arise from this way of thinking, but first the hypothesis needs testing. A testable prediction is that any emergent community-level effect of HGT will depend on the diversity of interacting components. For example, a community-level process dependent on a network of genetic interactions is likely to require the number of interacting partners—and thus combinatorial possibilities—to exceed a threshold level. If so, then one way to explore its existence would be to manipulate resource availability and measure the functional impact of ensuing changes in diversity on the presumed community-level process.

## Data accessibility

This article has no additional data.

## References

[1] Tringe SG et al. 2005 Comparative metagenomics of microbial communities. Science 308, 554–557. (10.1126/science.1107851) 15845853

[2] The Human Microbiome Project Consortium 2012 Structure, function and diversity of the healthy human microbiome. Nature 486, 207–214 (10.1038/nature11234) 22699609 PMC3564958

[3] Schloss PD, Handelsman J 2006 Toward a census of bacteria in soil. PLoS Comput. Biol. 2, e92 (10.1371/journal.pcbi.0020092) 16848637 PMC1513271

[4] Arnoldini M, Cremer J, Hwa T 2018 Bacterial growth, flow, and mixing shape human gut microbiota density and composition. Gut Microbes 9, 559–566. (10.1080/19490976.2018.1448741) 29533125 PMC6287699

[5] Whitman WB, Coleman DC, Wiebe WJ 1998 Prokaryotes: the unseen majority. Proc. Natl Acad. Sci. USA 95, 6578–6583. (10.1073/pnas.95.12.6578) 9618454 PMC33863

[6] Tierney BT, Yang Z, Luber JM, Beaudin M, Wibowo MC, Baek C, Mehlenbacher E, Patel CJ, Kostic AD 2019 The landscape of genetic content in the gut and oral human microbiome. Cell Host Microbe 26, 283–295.e8. (10.1016/j.chom.2019.07.008) 31415755 PMC6716383

[7] Schimel JP, Schaeffer SM 2012 Microbial control over carbon cycling in soil. Front. Microbiol. 3, 348 (10.3389/fmicb.2012.00348) 23055998 PMC3458434

[8] Zak JC, Willig MR, Moorhead DL, Wildman HG 1994 Functional diversity of microbial communities: a quantitative approach. Soil Biol. Biochem. 26, 1101–1108. (10.1016/0038-0717(94)90131-7)

[9] Hibbing ME, Fuqua C, Parsek MR, Peterson SB 2010 Bacterial competition: surviving and thriving in the microbial jungle. Nat. Rev. Microbiol. 8, 15–25. (10.1038/nrmicro2259) 19946288 PMC2879262

[10] Liu J, Martinez-Corral R, Prindle A, Lee D-yD, Larkin J, Gabalda-Sagarra M, Garcia-Ojalvo J, Süel GM 2017 Coupling between distant biofilms and emergence of nutrient time-sharing. Science 356, 638–642. (10.1126/science.aah4204) 28386026 PMC5645014

[11] Hendry AP 2017 Eco-evolutionary dynamics. Princeton, NJ: Princeton University Press.

[12] Hansen SK, Rainey PB, Haagensen JA, Molin S 2007 Evolution of species interactions in a biofilm community. Nature 445, 533–536. (10.1038/nature05514) 17268468

[13] Wang Q, Garrity GM, Tiedje JM, Cole JR 2007 Naive Bayesian classifier for rapid assignment of rRNA sequences into the new bacterial taxonomy. Appl. Environ. Microbiol. 73, 5261–5267. (10.1128/Aem.00062-07) 17586664 PMC1950982

[14] Vetrovsky T, Baldrian P 2013 The variability of the 16S rRNA gene in bacterial genomes and its consequences for bacterial community analyses. PLoS ONE 8, e57923 (10.1371/journal.pone.0057923) 23460914 PMC3583900

[15] Haruta S, Yamamoto K 2018 Model microbial consortia as tools for understanding complex microbial communities. Curr. Genom. 19, 723–733. (10.2174/1389202919666180911131206) PMC6225455 30532651

[16] Vetsigian K 2017 Diverse modes of eco-evolutionary dynamics in communities of antibiotic-producing microorganisms. Nat. Ecol. Evol. 1, 0189 (10.1038/s41559-017-0189)

[17] Sanchez-Gorostiaga A, Bajic D, Osborne ML, Poyatos JF, Sanchez A 2019 High-order interactions distort the functional landscape of microbial consortia. PLoS Biol. 17, e3000550 (10.1371/journal.pbio.3000550) 31830028 PMC6932822

[18] Vetsigian K, Jajoo R, Kishony R 2011 Structure and evolution of Streptomyces interaction networks in soil and in silico. PLoS Biol. 9, e1001184 (10.1371/journal.pbio.1001184) 22039352 PMC3201933

[19] Maynard Smith J. 1995 Do bacteria have population genetics? In Soc. Gen. Microbiol. Symp. 52. Population genetics of bacteria (eds Baumberg S, Young JPW, Wellington EMH, Saunders JR), pp. 1–12. Cambridge, UK: Cambridge University Press.

[20] Lässig M, Mustonen V, Walczak AM 2017 Predicting evolution. Nat. Ecol. Evol. 1, 77 (10.1038/s41559-016-0027) 28812721

[21] Hanage WP 2016 Not so simple after all: bacteria, their population genetics, and recombination. Cold Spring Harb. Perspect. Biol. 8, 1–18. (10.1101/cshperspect.a018069) PMC4930924 27091940

[22] Verster AJ, Ross BD, Radey MC, Bao YQ, Goodman AL, Mougous JD, Borenstein E 2017 The landscape of type VI secretion across human gut microbiomes reveals its role in community composition. Cell Host Microbe 22, 411–419.e4. (10.1016/j.chom.2017.08.010) 28910638 PMC5679258

[23] Bacci G et al. 2017 A different microbiome gene repertoire in the airways of cystic fibrosis patients with severe lung disease. Int. J. Mol. Sci. 18, 1654 (10.3390/ijms18081654) PMC5578044 28758937

[24] Karimi E, Ramos M, Goncalves JMS, Xavier JR, Reis MP, Costa R 2017 Comparative metagenomics reveals the distinctive adaptive features of the *Spongia officinalis* endosymbiotic consortium. Front. Microbiol. 8, 2499 (10.3389/fmicb.2017.02499) 29312205 PMC5735121

[25] Bonilla-Rosso G, Peimbert M, Alcaraz LD, Hernandez I, Eguiarte LE, Olmedo-Alvarez G, Souza V 2012 Comparative metagenomics of two microbial mats at Cuatro Cienegas Basin II: community structure and composition in oligotrophic environments. Astrobiology 12, 659–673. (10.1089/ast.2011.0724) 22920516 PMC3426889

[26] Truong DT, Tett A, Pasolli E, Huttenhower C, Segata N 2017 Microbial strain-level population structure and genetic diversity from metagenomes. Genome Res. 27, 626–638. (10.1101/gr.216242.116) 28167665 PMC5378180

[27] Segata N, Waldron L, Ballarini A, Narasimhan V, Jousson O, Huttenhower C 2012 Metagenomic microbial community profiling using unique clade-specific marker genes. Nat. Methods 9, 811–814. (10.1038/Nmeth.2066) 22688413 PMC3443552

[28] Nayfach S, Rodriguez-Mueller B, Garud N, Pollard KS 2016 An integrated metagenomics pipeline for strain profiling reveals novel patterns of bacterial transmission and biogeography. Genome Res. 26, 1612–1625. (10.1101/gr.201863.115) 27803195 PMC5088602

[29] Li Y, Wang H, Nie K, Zhang C, Zhang Y, Wang J, Niu PH, Ma XJ 2016 VIP: an integrated pipeline for metagenomics of virus identification and discovery. Scient. Rep. 6, 23774 (10.1038/srep23774) PMC4824449 27026381

[30] Luo CW, Knight R, Siljander H, Knip M, Xavier RJ, Gevers D 2015 ConStrains identifies microbial strains in metagenomic datasets. Nat. Biotechnol. 33, 1045–1052. (10.1038/nbt.3319) 26344404 PMC4676274

[31] Garud NR, Good BH, Hallatschek O, Pollard KS 2019 Evolutionary dynamics of bacteria in the gut microbiome within and across hosts. PLoS Biol. 17, e3000102 (10.1371/journal.pbio.3000102) 30673701 PMC6361464

[32] Frazão N, Sousa A, Lässig M, Gordo I 2019 Horizontal gene transfer overrides mutation in *Escherichia coli* colonizing the mammalian gut. Proc. Natl Acad. Sci. USA 116, 17 906–17 915. (10.1073/pnas.1906958116) 31431529 PMC6731689

[33] Zhao SJ, Lieberman TD, Poyet M, Kauffman KM, Gibbons SM, Groussin M, Xavier RJ, Alm EJ 2019 Adaptive evolution within gut microbiomes of healthy people. Cell Host Microbe 25, 656–667. (10.1016/j.chom.2019.03.007) 31028005 PMC6749991

[34] Robinson CD, Klein HS, Murphy KD, Parthasarathy R, Guillemin K, Bohannan BJM 2018 Experimental bacterial adaptation to the zebrafish gut reveals a primary role for immigration. PLoS Biol. 16, e2006893 (10.1371/journal.pbio.2006893) 30532251 PMC6301714

[35] Doolittle WF, Booth A 2017 It's the song, not the singer: an exploration of holobiosis and evolutionary theory. Biol. Philos. 32, 5–24. (10.1007/s10539-016-9542-2)

[36] Nicholls SM, Quick JC, Tang SQ, Loman NJ 2019 Ultra-deep, long-read nanopore sequencing of mock microbial community standards. GigaScience 8, giz043 (10.1093/gigascience/giz043) 31089679 PMC6520541

[37] Lawrence D, Fiegna F, Behrends V, Bundy JG, Phillimore AB, Bell T, Barraclough TG 2012 Species interactions alter evolutionary responses to a novel environment. PLoS Biol. 10, e1001330 (10.1371/journal.pbio.1001330) 22615541 PMC3352820

[38] Dekker J, Rippe K, Dekker M, Kleckner N 2002 Capturing chromosome conformation. Science 295, 1306–1311. (10.1126/science.1067799) 11847345

[39] Marbouty M, Koszul R 2015 Metagenome analysis exploiting high-throughput chromosome conformation capture (3C) data. Trends Genet. 31, 673–682. (10.1016/j.tig.2015.10.003) 26608779 PMC6831814

[40] Marbouty M, Cournac A, Flot JF, Marie-Nelly H, Mozziconacci J, Koszul R 2014 Metagenomic chromosome conformation capture (meta3C) unveils the diversity of chromosome organization in microorganisms. eLife 3, e03318 (10.7554/eLife.03318) 25517076 PMC4381813

[41] Marbouty M, Baudry L, Cournac A, Koszul R 2017 Scaffolding bacterial genomes and probing host-virus interactions in gut microbiome by proximity ligation (chromosome capture) assay. Sci. Adv. 3, e1602105 (10.1126/sciadv.1602105) 28232956 PMC5315449

[42] Yaffe E, Relman DA 2019 Tracking microbial evolution in the human gut using Hi-C reveals extensive horizontal gene transfer, persistence and adaptation. Nat. Microbiol. 5, 343–353. (10.1038/s41564-019-0625-0) 31873203 PMC6992475

[43] Morris JJ, Lenski RE, Zinser ER 2012 The Black Queen Hypothesis: evolution of dependencies through adaptive gene loss. mBio 3, e00036-12 (10.1128/mBio.00036-12) 22448042 PMC3315703

[44] Morris JJ, Zinser ER 2011 The Black Queen Hypothesis and the evolution of algal/bacterial mutualisms. J. Phycol. 47, S48.

[45] Fullmer MS, Soucy SM, Gogarten JP 2015 The pan-genome as a shared genomic resource: mutual cheating, cooperation and the black queen hypothesis. Front. Microbiol. 6, 728 (10.3389/fmicb.2015.00728) 26284032 PMC4523029

[46] Quistad SD, Doulcier G, Rainey PB 2020 Experimental manipulation of selfish genetic elements links genes to microbial community function. Phil. Trans. R. Soc. B 375, 20190681 (10.1098/rstb.2019.0681) 32200751 PMC7133536

[47] Elena SF, Lenski RE 2003 Evolution experiments with microorganisms: the dynamics and genetic bases of adaptation. Nat. Rev. Genet. 4, 457–469. (10.1038/nrg1088) 12776215

[48] Ochman H, Lawrence JG, Groisman EA 2000 Lateral gene transfer and the nature of bacterial innovation. Nature 405, 299–304. (10.1038/35012500) 10830951

[49] Silander OK, Weinreich DM, Wright KM, O'Keefe KJ, Rang CU, Turner PE, Chao L 2005 Widespread genetic exchange among terrestrial bacteriophages. Proc. Natl Acad. Sci. USA 102, 19 009–19 014. (10.1073/pnas.0503074102) PMC1323146 16365305

[50] McDonald MJ, Rice DP, Desai MM 2016 Sex speeds adaptation by altering the dynamics of molecular evolution. Nature 531, 233–236. (10.1038/nature17143) 26909573 PMC4855304

[51] Goddard MR, Godfray HCJ, Burt A 2005 Sex increases the efficacy of natural selection in experimental yeast populations. Nature 434, 636–640. (10.1038/nature03405) 15800622

[52] Doolittle WF, Sapienza C 1980 Selfish genes, the phenotype paradigm and genome evolution. Nature 284, 601–603. (10.1038/284601a0) 6245369

[53] Orgel LE, Crick FHC 1980 Selfish DNA: the ultimate parasite. Nature 284, 604–607. (10.1038/284604a0) 7366731

[54] Bergstrom CT, Lipsitch M, Levin BR 2000 Natural selection, infectious transfer and the existence conditions for bacterial plasmids. Genetics 155, 1505–1519. 10924453 10.1093/genetics/155.4.1505 PMC1461221

[55] Burt A, Trivers RL 2006 Genes in conflict: the biology of selfish genetic elements. Cambridge, MA: Belknap Press.

[56] Casjens S 2003 Prophages and bacterial genomics: what have we learned so far? Mol. Microbiol. 49, 277–300. (10.1046/j.1365-2958.2003.03580.x) 12886937

[57] Bobay LM, Touchon M, Rocha EP 2014 Pervasive domestication of defective prophages by bacteria. Proc. Natl Acad. Sci. USA 111, 12 127–12 132. (10.1073/pnas.1405336111) PMC4143005 25092302

[58] Cottier F, Srinivasan KG, Yurieva M, Liao W, Poidinger M, Zolezzi F, Pavelka N 2018 Advantages of meta-total RNA sequencing (MeTRS) over shotgun metagenomics and amplicon-based sequencing in the profiling of complex microbial communities. npj Biofilms Microbiomes 4, 2 (10.1038/s41522-017-0046-x) 29367879 PMC5773663

[59] Levy SF, Blundell JR, Venkataram S, Petrov DA, Fisher DS, Sherlock G 2015 Quantitative evolutionary dynamics using high-resolution lineage tracking. Nature 519, 181–186. (10.1038/nature14279) 25731169 PMC4426284

[60] Wilson DS, Sober E 1989 Reviving the superorganism. J. Theor. Biol. 136, 337–356. (10.1016/S0022-5193(89)80169-9) 2811397

[61] Rainey PB, Remigi P, Farr AD, Lind PA 2017 Darwin was right: where now for experimental evolution? Curr. Opin Genet. Dev. 47, 102–109. (10.1016/j.gde.2017.09.003) 29059583

[62] Xie L, Yuan AE, Shou WY 2019 Simulations reveal challenges to artificial community selection and possible strategies for success. PLoS Biol. 17, e3000295 (10.1371/journal.pbio.3000295) 31237866 PMC6658139

[63] Black AJ, Bourrat P, Rainey PB 2020 Ecological scaffolding and the evolution of individuality. Nat. Ecol. Evol. 4 (10.1038/s41559-019-1086-9) 32042121

[64] Swenson W, Arendt J, Wilson DS 2000 Artificial selection of microbial ecosystems for 3-chloroaniline biodegradation. Environ. Microbiol. 2, 564–571. (10.1046/j.1462-2920.2000.00140.x) 11233164

[65] Swenson W, Wilson DS, Elias R 2000 Artificial ecosystem selection. Proc. Natl Acad. Sci. USA 97, 9110–9114. (10.1073/pnas.150237597) 10890915 PMC16830

[66] Williams HTP, Lenton TM 2007 Artificial selection of simulated microbial ecosystems. Proc. Natl Acad. Sci. USA 104, 8918–8923. (10.1073/pnas.0610038104) 17517642 PMC1885603

[67] Doulcier G, Lambert AJ, De Monte S, Rainey PB 2019 Eco-evolutionary dynamics of nested Darwinian populations and the emergence of community-level heredity. bioRxiv 10, 827592 (10.1101/827592) PMC7440921 32633717

[68] Goodnight CJ. 2000 Heritability at the ecosystem level. Proc. Natl Acad. Sci. USA 17, 9365–9366. (10.1073/pnas.97.17.9365) PMC34031 10944208

[69] Hammerschmidt K, Rose C, Kerr B, Rainey PB 2014 Life cycles, fitness decoupling and the evolution of multicellularity. Nature 515, 75–79. (10.1038/nature13884) 25373677

[70] Lewontin RC 1970 The units of selection. Annu. Rev. Ecol. Syst. 1, 1–18. (10.1146/annurev.es.01.110170.000245)

[71] Godfrey-Smith P 2009 Darwinian populations and natural selection. Oxford, UK: Oxford University Press.

[72] Arias-Sánchez FI, Vessman B, Mitri S 2019 Artificially selecting microbial communities: if we can breed dogs, why not microbiomes? PLoS Biol. 17, e3000356 (10.1371/journal.pbio.3000356) 31469824 PMC6716632

[73] Rainey PB, De Monte S 2014 Resolving conflicts during the evolutionary transition to multicellular life. Annu. Rev. Ecol. Evol. Syst. 45, 599–620. (10.1146/annurev-ecolsys-120213-091740)

[74] Doolittle WF, Inkpen SA 2018 Processes and patterns of interaction as units of selection: an introduction to ITSNTS thinking. Proc. Natl Acad. Sci. USA 115, 4006–4014. (10.1073/pnas.1722232115) 29581311 PMC5910863

